# HOXA5 Suppresses NEK7‐Mediated Alveolar Epithelial Pyroptosis in Acute Lung Injury by Transcriptionally Inhibiting KAT2A

**DOI:** 10.1002/kjm2.70109

**Published:** 2025-09-25

**Authors:** Lei Wang, Heng Fan, Qi Wang, Cheng‐Jie Jiang, Dan‐Hui Li, Ji‐Hui Ye

**Affiliations:** ^1^ Department of Critical Care Medicine The First Affiliated Hospital of Ningbo University Ningbo People's Republic of China

**Keywords:** ALI, HOXA5, KAT2A, NEK7, Pyroptosis

## Abstract

Alveolar epithelial cell pyroptosis exacerbates inflammation and tissue damage by releasing inflammatory mediators, thereby promoting the development of acute lung injury (ALI). However, the fundamental mechanism underlying alveolar epithelial cell pyroptosis in ALI has not yet been elucidated. Lipopolysaccharide (LPS) was used to simulate ALI in vitro and in vivo. Cell viability was measured using the MTT assay. The expression of these molecules was determined by Western blot, enzyme‐linked immunosorbent assay (ELISA), quantitative real‐time polymerase chain reaction (RT‐qPCR), immunofluorescence, and immunohistochemical assays. The level of pyroptosis was determined using flow cytometry. The interactions between the molecules were validated using co‐immunoprecipitation, chromatin immunoprecipitation, and luciferase reporter assays. Homeobox A5 (HOXA5) was expressed at low levels, whereas lysine acetyltransferase 2A (KAT2A) and NIMA‐related kinase 7 (NEK7) were highly expressed in LPS‐induced (type II alveolar epithelial) ATII cells and mice. HOXA5 overexpression suppressed pyroptosis in LPS‐induced ATII cells and mice. Notably, KAT2A overexpression abolished the effects induced by HOXA5 overexpression in LPS‐induced ATII cells. Mechanistically, HOXA5 inhibits KAT2A transcriptional activity by binding to the KAT2A promoter. KAT2A positively regulates NEK7 by promoting H3K9ac/H3K27ac enrichment in the NEK7 promoter. In conclusion, HOXA5 indirectly inhibits NEK7 expression by inhibiting KAT2A transcriptional activity, thereby suppressing pyroptosis in alveolar epithelial cells in ALI.

**Abbreviations**
ALI: acute lung injury
ATII: type II alveolar epithelial
BSA: bovine serum albumin
ChIP: chromatin immunoprecipitation
Co‐IP: Co‐immunoprecipitation
DAB: diaminobenzidine
ELISA: enzyme‐linked immunosorbent assay
FBS: fetal bovine serum
H3K27ac: the acetylation of lysine at position 27 of histone H3
H3K36ac: the acetylation of lysine at position 36 of histone H3
H3K9ac: the acetylation of lysine at position 9 of histone H3
H4K8ac: the acetylation of lysine at position 8 of histone H4
HE: hematoxylin and eosin
HOXA5: Homeobox A5
IF: Immunofluorescence
IHC: Immunohistochemical
KAT2A: lysine acetyltransferase 2A
LPS: lipopolysaccharide
MTT: 3‐(4,5‐dimethylthiazol‐2‐yl)‐2,5‐diphenyltetrazolium bromide
NEK7: NIMA‐related kinase 7
NLRP3: NOD‐like receptor family pyrin domain containing 3
RT‐qPCR: quantitative real‐time polymerase chain reaction
SD: standard deviation
W/D: wet/dry

## 1 Introduction

Acute lung injury (ALI) is characterized by the impairment of pulmonary capillary endothelial cells and alveolar epithelial cells when factors such as severe infection, shock, and trauma are present, triggering noncardiogenic pulmonary edema and leading to progressive hypoxemia and dyspnea [1]. ALI, a critical condition in clinical settings, is characterized by sudden onset and a notably high mortality rate [2]. The current treatment strategies for this condition primarily involve managing the underlying disease, providing respiratory support, and implementing drug‐based therapies. Nevertheless, the absence of targeted medications and specialized approaches poses challenges, often preventing optimal therapeutic outcomes [3]. Therefore, investigating the pathogenesis of lung tissue injury in ALI and exploring novel therapeutic targets is of significant theoretical and practical value. Recent studies have revealed that pyroptosis, a novel modality of programmed inflammatory cell death, is intricately associated with the pathological progression of ALI [4, 5]. Therefore, we investigated the molecular mechanisms underlying pyroptosis in ALI alveolar epithelial cells.

Homeobox A5 (HOXA5), a member of the HOX gene family, displays abnormal expression in numerous diseases, including septic acute kidney injury and ALI [6, 7, 8]. For instance, HOXA5 protein levels declined in the pulmonary epithelial cell line (MLE‐12) after lipopolysaccharide (LPS) treatment, and HOXA5 overexpression alleviated ALI by suppressing ferroptosis in pulmonary epithelial cells [8], suggesting that HOXA5 is closely associated with the progression of ALI. Notably, Tang et al. reported that HOXA5 inhibits macrophage pyroptosis in osteoporosis [9]. There is currently a paucity of research on the role of HOXA5 in ALI. However, whether HOXA5 participates in the regulation of ALI progression by influencing pyroptosis in alveolar epithelial cells remains unknown.

NIMA‐related kinase 7 (NEK7), an integral member of the NIMA family, serves as a crucial regulator of multiple cellular processes [10]. NEK7 precisely regulates the cell cycle, actively engages in DNA damage repair mechanisms, facilitates intracellular protein transport, and modulates inflammatory responses [11]. At present, there is evidence that NEK7 directly interacts with NOD‐like receptor family pyrin domain containing 3 (NLRP3), thereby activating the NLRP3 inflammasome [12, 13], suggesting NEK7 regulates cell pyroptosis in diseases. In ALI, Britannin effectively impeded the interaction between NLRP3 and NEK7, thereby inhibiting the NLRP3 inflammasome [14]. Notably, in LPS‐induced ALI, NEK7 expression is enhanced in alveolar macrophages with LPS treatment, and decreasing NEK7 expression weakens the interaction between NEK7 and NLRP3, thus suppressing pyroptosis of alveolar macrophages in ALI [15]. In addition, a previous study reported that NEK7 expression was upregulated in the lung tissue of rats with ALI induced by sepsis, and that melatonin could mitigate septicemia‐associated ALI by decreasing NEK7 and Toll‐like receptor 2 expression [16]. The above‐mentioned evidence suggests that NEK7 is abnormally highly expressed in ALI and participates in the activation of the NLRP3 inflammasome through interaction with NLRP3, thus accelerating ALI progression by promoting pyroptosis. However, the mechanisms underlying NEK7 overexpression in ALI and its upstream regulation remain unknown and warrant further study.

HOXA5 functions as a transcription factor. It attaches to the promoters of genes, where it can either suppress or promote the transcription and expression of target genes [17, 18]. In this study, we found that HOXA5 regulates NEK7 expression in an indirect and negative manner. Specifically, HOXA5 inhibited lysine acetyltransferase 2A (KAT2A) expression by impairing the transcriptional activity of KAT2A, and KAT2A positively regulated NEK7 expression by recruiting lysine acetylation at position 9 of histone H3 (H3K9ac)/lysine acetylation at position 27 of histone H3 (H3K27ac) enrichment in the NEK7 promoter. Generally speaking, we found that HOXA5 negatively regulates the KAT2A/NEK7/NLRP3 axis to suppress pyroptosis in LPS‐induced ALI.

## 2 Methods

### 2.1 Cell Culture and Treatment

Human type II alveolar epithelial (ATII) cells were purchased from ProCell Co. Ltd. (Hubei, China). Cells were cultured in DMEM (Gibco, Carlsbad, CA, USA) supplemented with 10% fetal bovine serum (FBS; Gibco), 100 U/mL penicillin, and 100 mg/mL streptomycin (Beyotime, Jiangsu, China). Cells were maintained at 37°C in an environment with a 5% CO₂ atmosphere. To establish an in vitro cell model of ALI, ATII cells were incubated with 10 μg/mL LPS (Invitrogen, Carlsbad, CA, USA) for 24 h.

### 2.2 Cell Transfection

Overexpression plasmids for HOXA5 (oe‐HOXA5), NEK7 (oe‐NEK7), KAT2A (oe‐KAT2A), and oe‐NC were obtained from GenePharma (Shanghai, China). ATII cells were transfected with the aforementioned plasmids for 48 h using Lipofectamine 3000 (Invitrogen).

### 2.3 3‐(4,5‐Dimethylthiazol‐2‐Yl)‐2,5‐Diphenyltetrazolium Bromide (MTT) Assay

Cells were seeded in a 96‐well plate (5000 cells per well) and cultured for 24 h. Subsequently, 10 μL of MTT solution (Beyotime) was added to each well and incubated for 4 h. Then, the medium in each well was carefully aspirated. Next, 100 μL of DMSO (Beyotime) was added to dissolve the formazan crystals. The plate was shaken for 10 min. Finally, a microplate reader was used to measure the optical density at 570 nm.

### 2.4 Establishment of ALI Mouse Model

Male C57BL/6 mice (6–8 weeks old, 20–24 g) were obtained from the Laboratory Animal Center of Ningbo University. All animal experiments were approved by the Ethics Committee of Ningbo University in accordance with the relevant guidelines. Mice were maintained under standard SPF conditions: temperature 20 to 25°C, humidity 50%–60%, and a fixed light–dark cycle. One week later, to investigate the role of HOXA5 in ALI, the mice were randomly divided into four groups: control, LPS, LPS + oe‐NC, and LPS + oe‐HOXA5, with six mice in each group. To establish a mouse model of ALI, experiment adopted the LPS tracheal instillation method was used in this experiment. The specific procedures were as follows: a gas anesthetic machine and a small animal ventilator were used to administer continuous gas anesthesia and mechanical ventilation to the mice. Mice were anesthetized by inhalation of isoflurane at a concentration of 1%, followed by tracheal intubation. Then, LPS (5 mg/kg) dissolved in 50 μL of PBS was instilled into the trachea to induce ALI. Additionally, to overexpress HOXA5 in the lung tissues, a lentivirus‐packaged HOXA5 overexpression vector (5 × 10^7^ UT/mL, 5 μL; GenePharma) was injected intratracheally 5 days before LPS tracheal instillation. The mice were euthanized, and samples were collected 24 h after modeling.

### 2.5 Western Blot

ATII cells with different transfection treatments and lung tissues from mice were lysed using RIPA buffer (Beyotime). Protein concentrations were determined using a BCA kit (Beyotime), and equal amounts of protein were loaded into the wells of the gels, and the proteins were separated via electrophoresis. Separated proteins were transferred onto PVDF membranes. The PVDF membranes were incubated with 5% skimmed milk for 1 h. Primary antibodies against HOXA5 (PA5‐69008, 1:500, Thermo Fisher Scientific, Waltham, MA, USA), NEK7 (MA5‐47007, 1:5000, Thermo Fisher Scientific), KAT2A (MA5‐14884, 1:500, Thermo Fisher Scientific), NLRP3 (MA5‐32255, 1:1000, Thermo Fisher Scientific), ASC (PA5‐50915, 1:250, Thermo Fisher Scientific), Cleaved Caspase‐1 (PA5‐99390, 1:1000, Thermo Fisher Scientific), Cleaved GSDMD (ab215203, 1:1000, Abcam, Cambridge, UK; 10,137, CST, Danvers, MA, USA), and β‐actin (MA1‐140, 1:10000, Thermo Fisher Scientific) were incubated with the PVDF membranes overnight at 4°C. The following day, the PVDF membranes were washed three times with TBST solution, and secondary antibodies (Thermo Fisher Scientific) were incubated with the PVDF membranes for 2 h. Finally, ECL chemiluminescent reagent (Thermo Fisher Scientific) was used to visualize the protein bands, and the grayscale values were quantified using ImageJ.

### 2.6 Enzyme‐Linked Immunosorbent Assay (ELISA)

ATII cells were subjected to the indicated treatments and centrifuged to obtain cell supernatants. Bronchoalveolar lavage fluid was also collected from mice. Subsequently, IL‐1β and IL‐18 levels in the samples were measured using ELISA kits (PI305, PI558, Beyotime). After treatment, the absorbance of each well was measured at 450 nm according to the manufacturer's instructions.

### 2.7 RNA Isolation and Quantitative Real‐Time Polymerase Chain Reaction (RT‐qPCR)

Total RNA was extracted from ATII cells transfected with various plasmids using TRIzol reagent (Takara, Tokyo, Japan). Subsequently, an RNA reverse transcription kit (Takara) was used to reverse transcribe the total RNA into cDNA. SYBR Premix Ex Taq (Takara) was used to conduct quantitative real‐time PCR (qRT‐PCR) on an ABI Prism 7500 RT‐PCR system (Applied Biosystems). The relative expression of the genes was normalized to β‐actin using the 2^−ΔΔCt^ formula. The primer sequences are listed in Table 1.

**TABLE 1:** Primer information.

	Forward primers	Reverse primers
HOXA5	5′‐GCGCAAGCTGCACATAAGTC‐3′	5′‐CGGAGAGGCAAAGAGCATGT‐3′
NEK7	5′‐CCACTGGGGTGGTAAAACTTG‐3′	5′‐AAGGACTTTGTAATGCAGCCAT‐3′
KAT2A	5′‐TGAGGACGTGGCTACCTACAA‐3′	5′‐GGCGGGTAACGGTGAAAAT‐3′
β‐Actin	5′‐CCCTGGAGAAGAGCTACGAG −3′	5′‐CGTACAGGTCTTTGCGGATG −3′

Abbreviations: HOXA5, Homeobox A5; KAT2A, lysine acetyltransferase 2A; NEK7, NIMA‐related kinase 7.

### 2.8 Flow Cytometry

Pyroptosis in ATII cells was assessed using flow cytometry. Briefly, cells were collected, and suspensions were obtained. The FAM‐FLICA Caspase‐1 YVAD Assay Kit (ICT‐97, ImmunoChemistry, Bloomington, MN, USA) was used to detect cell pyroptosis. Cells were incubated with FAM‐YVAD‐FMK and stained with PI for 60 min. Cell pyroptosis was analyzed using a flow cytometer (BD, Franklin Lake, NJ, USA).

### 2.9 Co‐Immunoprecipitation (Co‐IP)

The Pierce Co‐IP Kit (26,149, Thermo Fisher Scientific) was used to validate the interaction between NEK7 and NLRP3. First, cell lysates were collected and precleared with Protein A/G beads to remove nonspecific proteins. Twenty percent of the lysate was used as the input control. The remaining lysates were incubated overnight at 4°C with anti‐NEK7 (A302‐684A, Thermo Fisher Scientific) or IgG (ab172730, Abcam), followed by capture using Protein A/G beads. After washing to remove unbound proteins, the complexes were eluted, denatured, and analyzed by Western blot.

### 2.10 Chromatin Immunoprecipitation (ChIP) Assay

Following the manufacturer's instructions, the EZ‐ChIP kit (17‐10461, Sigma‐Aldrich, St. Louis, MO, USA) was used for the ChIP assay. Briefly, cells were lysed and sonicated for 5 min after crosslinking with 1% formaldehyde. The lysates were then incubated overnight at 4°C with protein A/G Sepharose preincubated with anti‐H3K9ac (ab32129, Abcam), anti‐H3K27ac (MA5‐23516, Thermo Fisher Scientific), anti‐H4K8ac (701,796, Thermo Fisher Scientific), and anti‐HOXA5 (sc‐365,784, Santa Cruz, Dallas, TX, USA). Finally, after washing and eluting the beads, the precipitated DNA was purified and analyzed by qPCR.

### 2.11 Luciferase Reporter Assay

A luciferase reporter assay validated the interaction between the HOXA5 and KAT2 promoters. First, the KAT2A promoter was subcloned into the pGL3.0‐basic vector to construct pGL3‐KAT2A‐wildtype (WT) and pGL3‐KAT2A‐mutant (MUT). ATII cells were cultured in 24‐well plates, and luciferase reporter constructs were co‐transfected with oe‐NC or oe‐HOXA5 using Lipofectamine 3000. After 48 h, relative luciferase activity was assessed using the Dual‐luciferase Reporter Gene Analysis System (Promega, Madison, WI, USA), calculated as the ratio of firefly luciferase activity to Renilla luciferase activity.

### 2.12 Immunofluorescence (IF)

ATII cells were fixed in 4% paraformaldehyde for 30 min and permeabilized with 0.3% Triton X‐100 for 1 h. The membranes were blocked for 30 min with 5% bovine serum albumin. The cells were incubated overnight at 4°C with anti‐NEK7 (PA5‐101861, Thermo Fisher Scientific), anti‐NLRP3 (MA5‐34969, Thermo Fisher Scientific) and Surfactant Protein A (SP‐A) (11850–1‐AP, Proteintech, Wuhan, China). Subsequently, the cells were incubated with secondary antibodies for 1 h. The nuclei of ATII cells were stained with DAPI for 5 min. Finally, stained cells were observed under a microscope (Zeiss, Germany).

### 2.13 Hematoxylin and Eosin (HE) Staining and Lung Injury Score

The lung tissues were fixed in 4% paraformaldehyde for 24 h. The fixed lung tissues were then processed for paraffin embedding. Paraffin blocks were sectioned to maximize exposure of the bronchial tree structures. Tissues were prepared as 4‐μm sections and stained with hematoxylin and eosin to assess lung injury severity. Two independent investigators, blinded to sample group identities, evaluated tissue sections using a preestablished scoring system [19].

### 2.14 Lung Index and Lung Wet/Dry (W/D) Ratio

Prior to sacrifice, the body weight of each mouse was recorded. The entire lung tissue was carefully excised from the thoracic cavity of each mouse and weighed. Pulmonary edema was evaluated using two key parameters: lung index and lung wet/dry (W/D) ratio. The lung index was determined by calculating the ratio of lung weight to total body weight (Lung/Whole Body). To obtain the dry weight of the lungs, the excised lung tissues were placed in an oven maintained at 60°C for approximately 48 h to ensure complete dehydration, after which they were reweighed. The wet‐to‐dry weight ratios of the lungs were calculated based on these measurements.

### 2.15 Immunohistochemical (IHC) Assay

Lung tissue specimens were fixed in 4% paraformaldehyde and embedded in paraffin blocks. These blocks were then sectioned into 5 μm‐thick slices. Following antigen retrieval, sections were blocked with 1% bovine serum albumin (BSA). Subsequently, sections were incubated overnight at 4°C with the anti‐HOXA5 (PA5‐69008, Thermo Fisher Scientific) and anti‐NLRP3 (MA5‐32255, Thermo Fisher Scientific). The next day, the sections were treated with an HRP‐conjugated secondary antibody (Thermo Fisher Scientific), and color development was performed using diaminobenzidine (DAB; Beyotime). Finally, the stained sections were observed and imaged under a light microscope (Nikon).

## 3 Statistical Analysis

Data were analyzed using GraphPad Prism 9.0. Data are presented as the mean ± standard deviation (SD). An unpaired Student's t‐test was used to compare two groups. For comparisons involving three or more groups, a one‐way ANOVA followed by Tukey's post hoc test was applied. The statistical significance threshold was set at *p* < 0.05. All data were obtained from three independent experiments.

## 4 Results

### 4.1 HOXA5 Overexpression Inhibited LPS‐Induced Pyroptosis of ATII Cells

**FIGURE 1:**
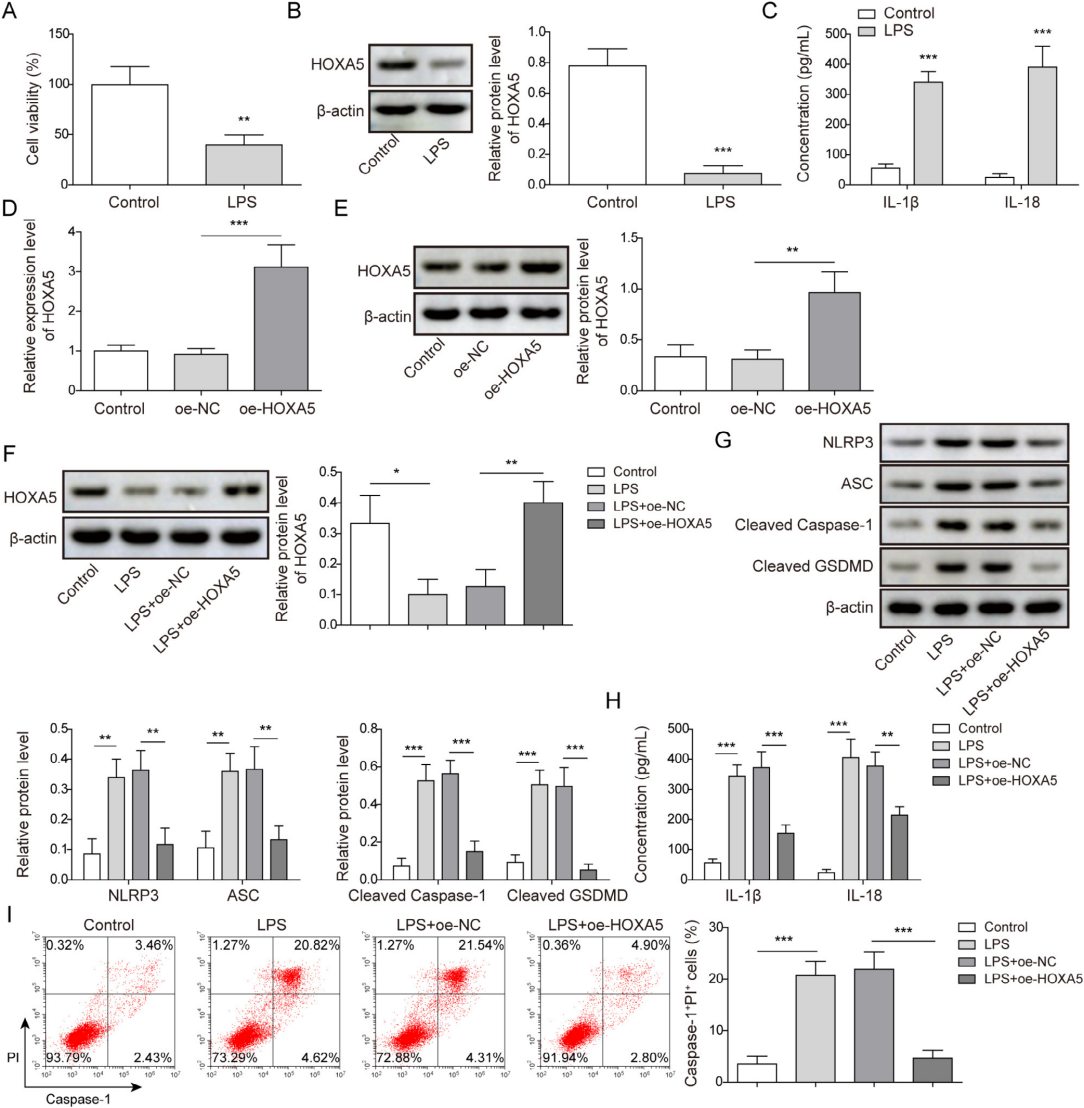
Homeobox A5 (HOXA5) overexpression inhibited lipopolysaccharide (LPS)‐induced pyroptosis of type II alveolar epithelial (ATII) cells. 10 μg/mL LPS was used for treating ATII cells for 24 h. (A) Cell viability was detected using 3‐(4,5‐dimethylthiazol‐2‐yl)‐2,5‐diphenyltetrazolium bromide (MTT) assay. (B) HOXA5 levels were determined using Western blot. (C) IL‐1β and IL‐18 levels in cell supernatant were examined using enzyme‐linked immunosorbent assay (ELISA). (D‐E) HOXA5 levels in ATII cells transfected with oe‐NC or oe‐HOXA5 were measured by quantitative real‐time polymerase chain reaction (RT‐qPCR) and Western blot. oe‐HOXA5 transfected ATII cells were then treated with LPS. (F) HOXA5 levels were determined by Western blot. (G) NLRP3, ASC, Cleaved Caspase‐1, and Cleaved GSDMD levels were detected using Western blot. (H) IL‐1β and IL‐18 levels in cell supernatant were examined using enzyme‐linked immunosorbent assay (ELISA). (I) The pyroptosis rate was investigated using flow cytometry. *p < 0.05, **p < 0.01, ***p < 0.001.

First, we used ATII cells to detect the role of HOXA5 in pyroptosis. The ATII cells were identified as alveolar type II cells by IF staining for SP‐A (a protein specifically synthesized and secreted by ATII). The detection results showed that the proportion of SP‐A‐positive cells exceeded 90% (Supplementary Figure S1). Then, the ATII cells were treated with 10 μg/mL LPS for 24 h. As shown in Figure 1A, LPS treatment significantly impaired the viability of ATII cells. Western blot revealed that LPS treatment resulted in abnormally low HOXA5 protein levels in ATII cells (Figure 1B). Moreover, LPS treatment led to elevated levels of cell pyroptosis‐related inflammatory factors (IL‐1β and IL‐18) in ATII cells (Figure 1C). To investigate the influence of HOXA5 on LPS‐induced pyroptosis in ATII cells, the ATII cells were transfected with oe‐HOXA5 to elevate HOXA5 expression (Figure 1D,E). In addition, LPS‐induced reduction in HOXA5 levels in ATII cells was abolished by oe‐HOXA5 transfection (Figure 1F). In terms of pyroptosis‐related indicators, LPS treatment promoted pyroptosis in ATII cells, as evidenced by elevated NLRP3, ASC, Cleaved Caspase‐1, Cleaved GSDMD, IL‐1β, IL‐18, and pyroptosis cells. However, HOXA5 overexpression abolished LPS‐induced pyroptosis (Figure 1G–I). Taken together, these results indicate that HOXA5 upregulation weakens LPS‐induced pyroptosis in ATII cells.

### 4.2 HOXA5 Reduced NEK7 Expression to Inactivate the NLRP3 Inflammasome

**FIGURE 2:**
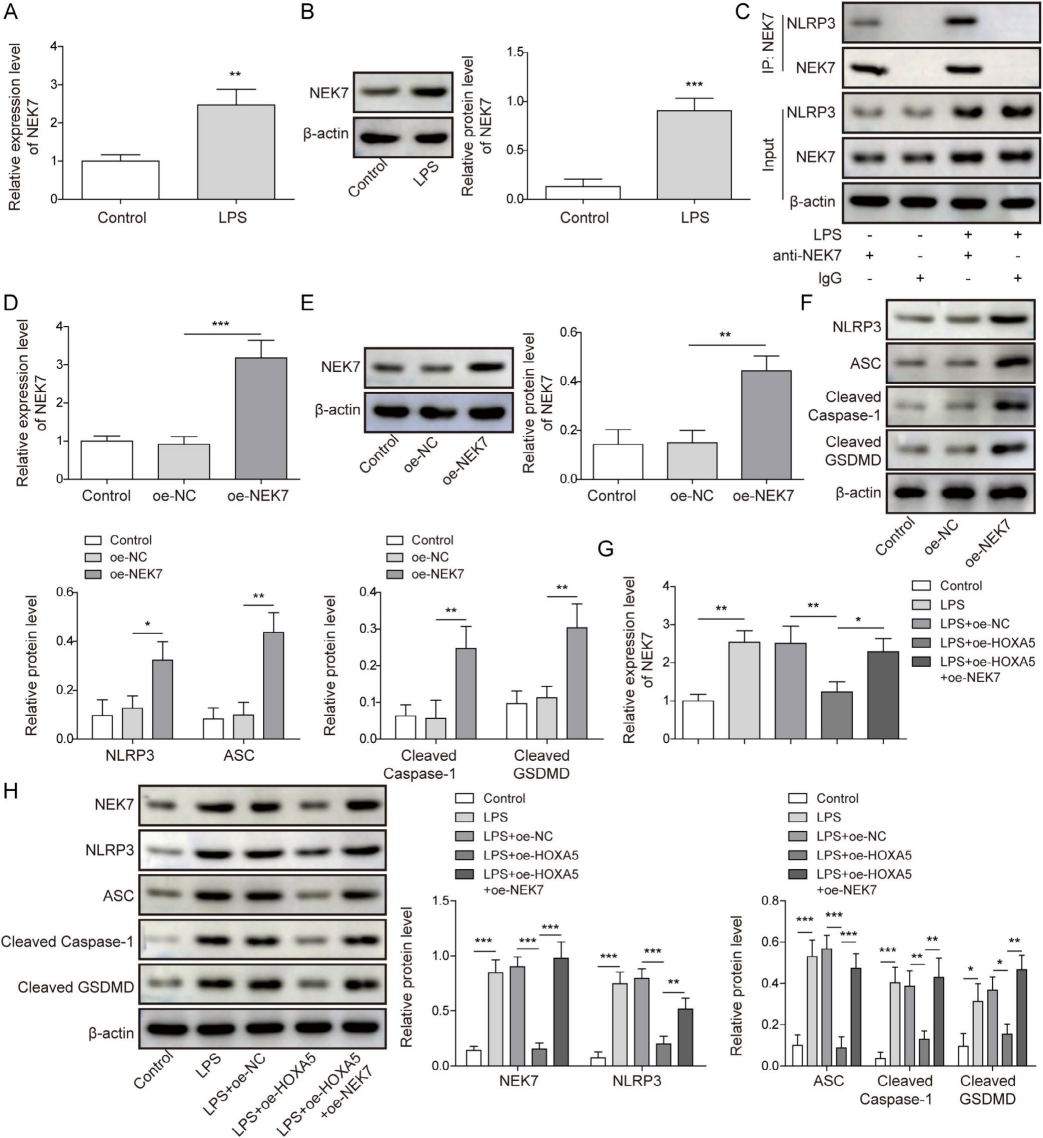
Homeobox A5 (HOXA5) reduced NEK7 expression to inactivate the NLRP3 inflammasome. (A, B) NEK7 expression in type II alveolar epithelial (ATII) cells with lipopolysaccharide (LPS) treatment was measured using quantitative real‐time polymerase chain reaction (RT‐qPCR) and Western blot. (C) The interaction between NEK7 and NLRP3 proteins in ATII cells with and without LPS treatment was validated by Co‐IP. ATII cells were transfected with oe‐NC or oe‐NEK7. (D, E) NEK7 expression was detected by RT‐qPCR and Western blot. (F) NLRP3, ASC, Cleaved Caspase‐1, and Cleaved GSDMD levels were detected by Western blot. ATII cells were transfected with either oe‐HOXA5 alone or in combination with oe‐NEK7, and subsequently treated with LPS. (G) NEK7 expression was measured by RT‐qPCR. (H) NEK7, NLRP3, ASC, Cleaved Caspase‐1, and Cleaved GSDMD levels were detected by Western blot. *p < 0.05, **p < 0.01, ***p < 0.001.

Previous studies have suggested that NEK7 is a key factor in regulating NLRP3‐mediated pyroptosis [20]. Interestingly, LPS induction enhanced NEK7 expression in ATII cells (Figure 2A,B). A previous study revealed that NEK7 interacts with NLRP3, thereby facilitating activation of the NLRP3 inflammasome [21]. Herein, we validated the interaction between NEK7 and NLRP3 proteins in ATII cells using a co‐IP assay and found that LPS treatment promoted this interaction (Figure 2C). Next, we overexpressed NEK7 in ATII cells using oe‐NEK7 transfection (Figure 2D,E). As expected, NEK7 overexpression elevated the levels of NLRP3, ASC, Cleaved Caspase‐1, and Cleaved GSDMD (Figure 2F). In addition, ATII cells were transfected with oe‐HOXA5 alone or in combination with oe‐NEK7, followed by LPS treatment. As presented in Figure 2G,H, HOXA5 upregulation offset the LPS‐induced increase in NEK7 expression in ATII cells, whereas NEK7 overexpression eliminated the effect of HOXA5 upregulation on NEK7 expression in LPS‐induced ATII cells. NEK7 overexpression also counteracted the HOXA5 upregulation‐induced reduction in NLRP3, ASC, Cleaved Caspase‐1, and Cleaved GSDMD in LPS‐induced ATII cells (Figure 2H). Collectively, HOXA5 upregulation suppresses pyroptosis in LPS‐induced ATII cells by reducing NEK7 expression.

### 4.3 KAT2A Raised NEK7 Expression via Promoting the Enrichment of H3K9ac/H3K27ac in NEK7 Promoter

**FIGURE 3:**
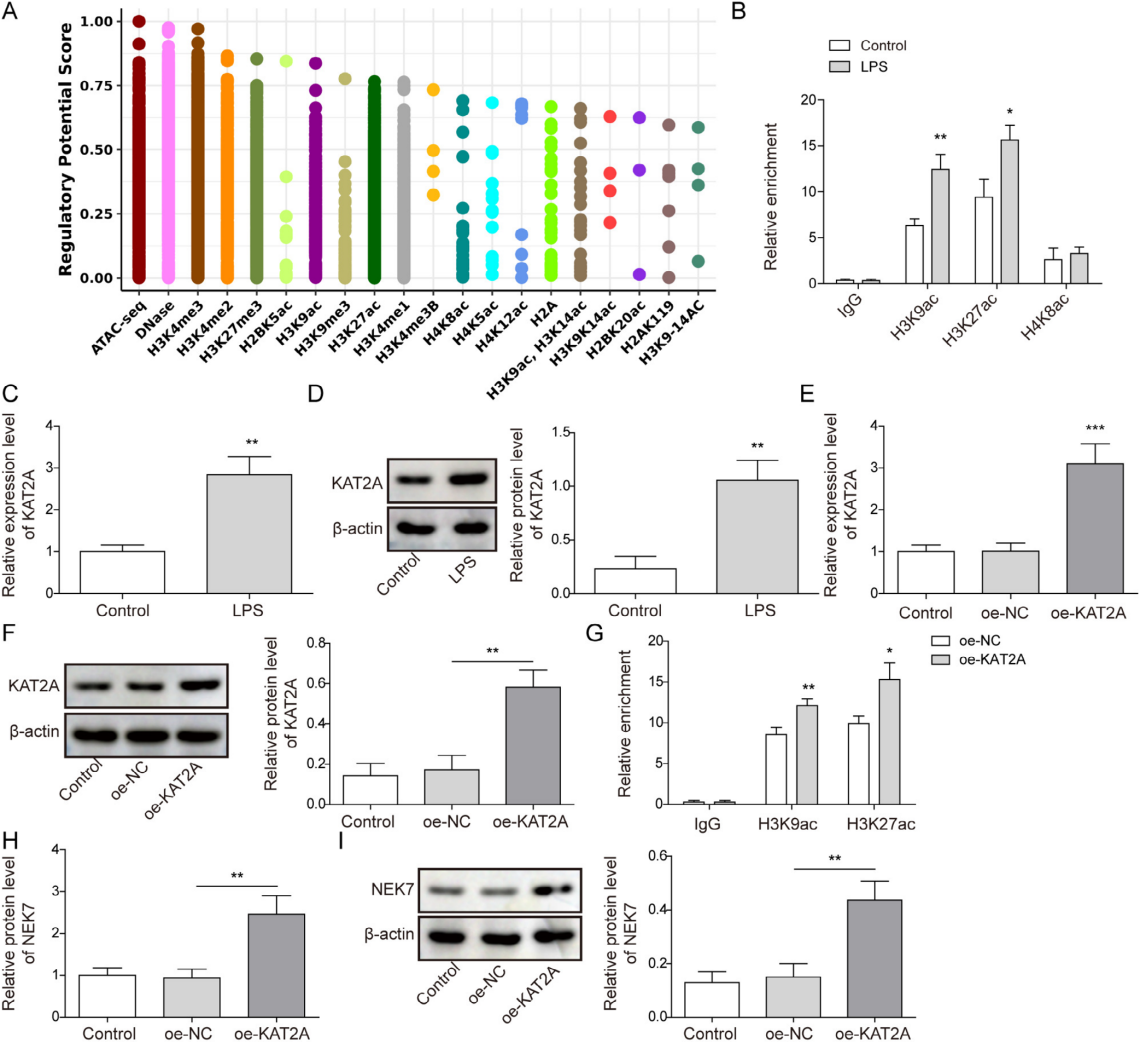
Lysine acetyltransferase 2A (KAT2A) raised NEK7 expression by promoting the enrichment of H3K9ac/H3K27ac in NEK7 promoter. (A) Multiple epigenetic modification marks in the NEK7 promoter region were predicted using the Cistrome database (http://cistrome.org/db/#/). Type II alveolar epithelial (ATII) cells were treated with 10 μg/mL lipopolysaccharide (LPS) for 24 h. (B) The enrichment of H3K9ac, H3K27ac, and H4K8ac in the NEK7 promoter region was determined using chromatin immunoprecipitation (ChIP). (C‐D) KAT2A expression was detected by quantitative real‐time polymerase chain reaction (RT‐qPCR) and Western blot. ATII cells were transfected with oe‐NC or oe‐KAT2A. (E‐F) KAT2A expression was detected by RT‐qPCR and Western blot. (G) The enrichment of H3K9ac and H3K27ac in the NEK7 promoter region was determined using ChIP. (H‐I) NEK7 expression was examined by RT‐qPCR and Western blot. *p < 0.05, **p < 0.01, ***p < 0.001.

Using the Cistrome database, we predicted the presence of multiple epigenetic modifications in the NEK7 promoter (Figure 3A). Furthermore, ChIP assay revealed the enrichment of H3K9ac, H3K27ac, and the acetylation of lysine at position 8 in histone H4 (H4K8ac) in the NEK7 promoter region. LPS treatment promoted the enrichment of H3K9ac and H3K27ac in this region (Figure 3B). Interestingly, KAT2A expression was elevated in ATII cells after LPS treatment (Figure 3C,D). Here, we overexpressed KAT2A in ATII cells using oe‐KAT2A transfection (Figure 3E,F). As expected, KAT2A overexpression significantly boosted the enrichment of H3K9ac and H3K27ac in the NEK7 promoter region (Figure 3G). Notably, KAT2A overexpression increased NEK7 expression (Figure 3H,I). Overall, KAT2A recruits H3K9ac/H3K27ac to the NEK7 promoter, thereby upregulating NEK7 expression.

### 4.4 HOXA5 Reduced the H3K9ac/H3K27ac Enrichment in NEK7 Promoter by Transcriptional Inhibition of KAT2A

**FIGURE 4:**
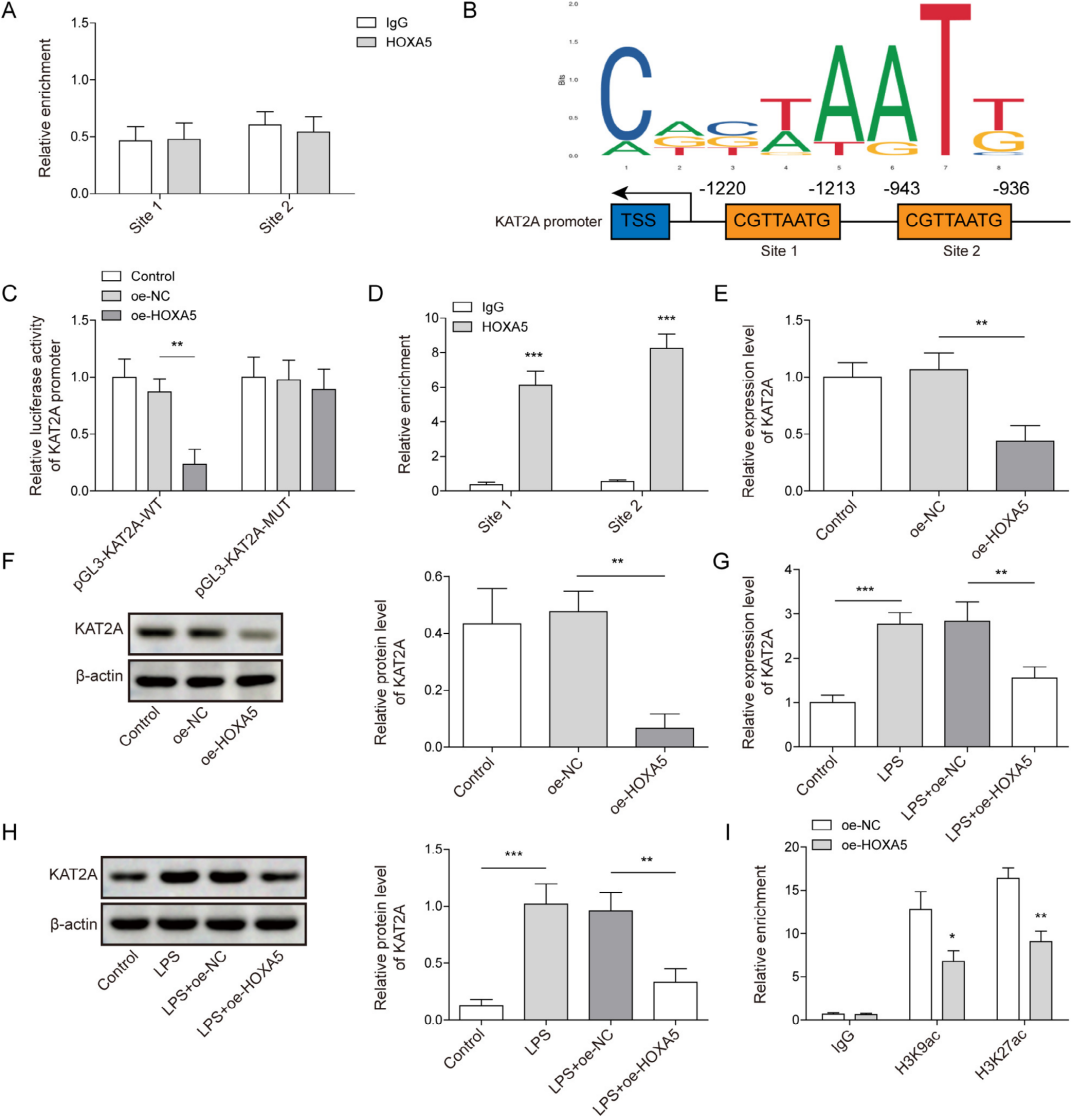
Homeobox A5 (HOXA5) lessened the H3K9ac/H3K27ac enrichment in NEK7 promoter by transcriptional inhibition of lysine acetyltransferase 2A (KAT2A). (A) The direct interaction between HOXA5 and NEK7 promoters in type II alveolar epithelial (ATII) cells was analyzed using chromatin immunoprecipitation (ChIP). (B) The binding sites between HOXA5 and KAT2A promoter were predicted using the JASPAR database. (C‐D) The interaction between HOXA5 and KAT2A promoters was validated using luciferase reporter assay and ChIP assay. (E, F) KAT2A expression in ATII cells with oe‐HOXA5 transfection was detected by quantitative real‐time polymerase chain reaction (RT‐qPCR) and Western blot. ATII cells with oe‐HOXA5 transfection were treated with LPS. (G‐H) KAT2A expression was examined by RT‐qPCR and Western blot. ATII cells were transfected with oe‐NC or oe‐HOXA5. (I) The enrichment of H3K9ac and H3K27ac in the NEK7 promoter region was determined using ChIP. *p < 0.05, **p < 0.01, ***p < 0.001.

As shown in Figure 2G,H, HOXA5 downregulated NEK7 expression in LPS‐induced ATII cells. Therefore, we examined whether the transcription factor HOXA5 directly interacts with the NEK7 promoter to influence NEK7 expression. As shown in Figure 4A, the ChIP assay indicated that HOXA5 did not directly bind to NEK7 promoter sites in ATII cells, suggesting that HOXA5 may indirectly regulate NEK7 expression in LPS‐induced ATII cells. We used the JASPAR database to predict the binding sites of the HOXA5 and KAT2A promoters (Figure 4B). The luciferase reporter gene experiment showed that HOXA5 overexpression inhibited luciferase activity in the pGL3‐KAT2A‐WT group but not in the pGL3‐KAT2A‐MUT (Figure 4C), suggesting an interaction between HOXA5 and the KAT2A promoter. Moreover, the interaction between HOXA5 and the KAT2A promoter was validated by the ChIP assay (Figure 4D). As shown in Figure 4E,F, HOXA5 overexpression reduced KAT2A expression in ATII cells. Additionally, KAT2A expression was elevated in ATII cells following LPS induction, which was attenuated by HOXA5 overexpression (Figure 4G,H). Additionally, HOXA5 overexpression impaired H3K9ac and H3K27ac enrichment in the NEK7 promoter region (Figure 4I). In summary, HOXA5 inhibited KAT2A transcriptional activity and KAT2A expression, which diminished the enrichment of H3K9ac and H3K27ac in the NEK7 promoter region.

### 4.5 HOXA5 Overexpression Inhibited Pyroptosis in LPS‐Induced ATII Cells Through Decreasing KAT2A Expression

**FIGURE 5:**
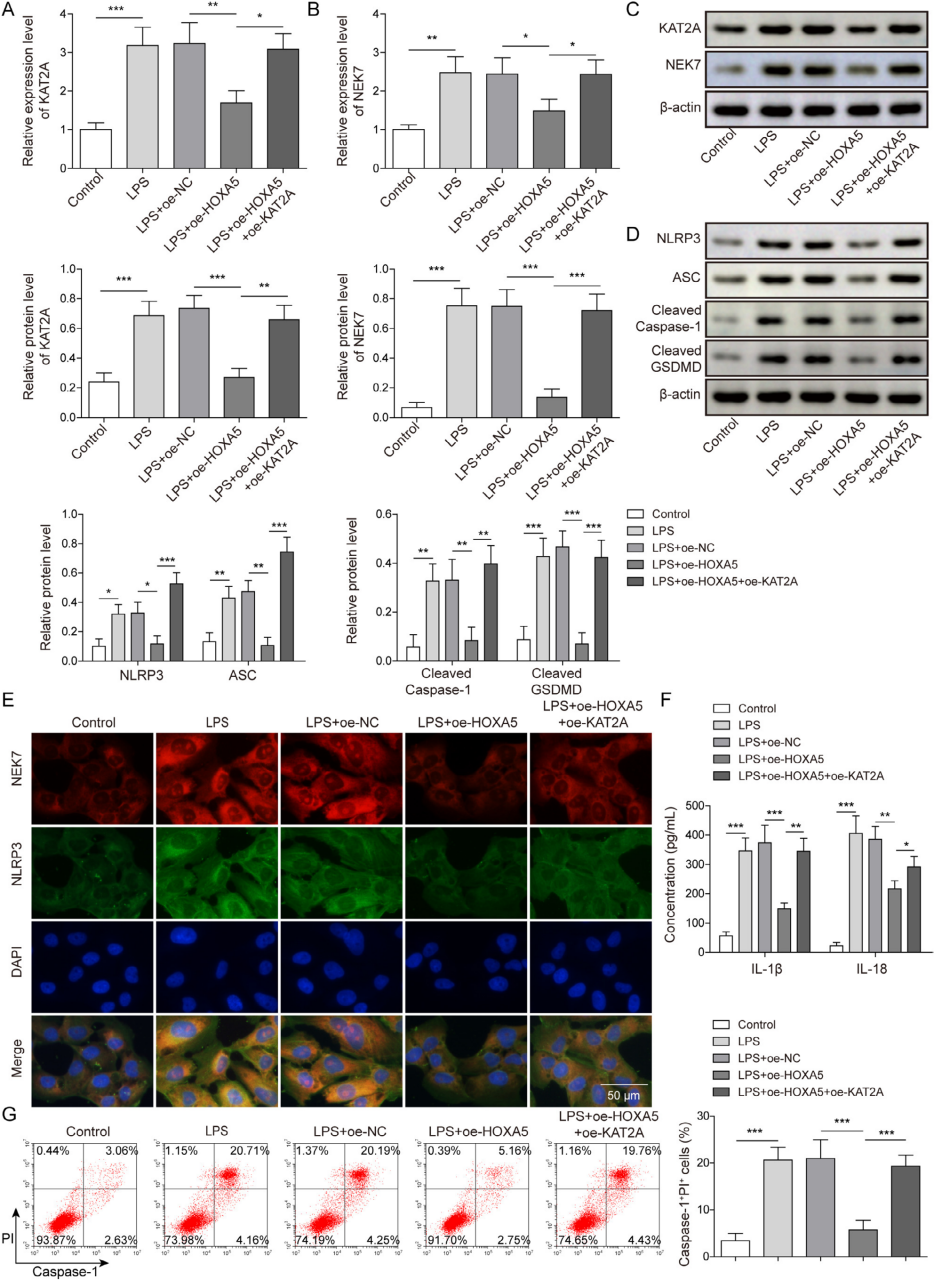
Homeobox A5 (HOXA5) overexpression inhibited pyroptosis in LPS‐induced type II alveolar epithelial (ATII) cells through decreasing lysine acetyltransferase 2A (KAT2A) expression. ATII cells were transfected with either oe‐HOXA5 alone or in combination with oe‐KAT2A and subsequently treated with lipopolysaccharide (LPS). (A‐C) KAT2A and NEK7 levels were measured by quantitative real‐time polymerase chain reaction (RT‐qPCR) and Western blot. (D) NLRP3, ASC, Cleaved Caspase‐1, and Cleaved GSDMD levels were detected using Western blot. (E) Co‐expression of NEK7 and NLRP3 was measured using IF assay. (F) IL‐1β and IL‐18 levels in cell supernatants were examined using enzyme‐linked immunosorbent assay (ELISA). (G) The pyroptosis rate was investigated using flow cytometry. *p < 0.05, **p < 0.01, ***p < 0.001.

We investigated the effect of the HOXA5/KAT2A axis on pyroptosis in LPS‐induced ATII cells. ATII cells were transfected with oe‐HOXA5 alone or with oe‐KAT2A, followed by LPS treatment. We observed that HOXA5 upregulation decreased KAT2A and NEK7 expression in LPS‐induced ATII cells. However, KAT2A overexpression reversed the reduction in KAT2A and NEK7 expression by HOXA5 upregulation (Figure 5A–C). Moreover, HOXA5 upregulation decreased the levels of NLRP3, ASC, Cleaved Caspase‐1, and Cleaved GSDMD in LPS‐induced ATII cells, and this reduction was partially reversed by KAT2A overexpression (Figure 5D). Using an IF assay, we observed that NEK7 and NLRP3 were co‐expressed in the cytoplasm of ATII cells. LPS treatment promoted the co‐expression of NEK7 and NLRP3, which was attenuated by HOXA5 overexpression. Notably, in LPS‐induced ATII cells, KAT2A overexpression reversed the HOXA5 overexpression‐mediated reduction in the co‐expression of NEK7 and NLRP3 (Figure 5E). As expected, the decrease in IL‐1β and IL‐18 levels by HOXA5 overexpression in LPS‐treated ATII cells was reversed by KAT2A overexpression (Figure 5F). Flow cytometry revealed that HOXA5 overexpression suppressed LPS‐induced upregulation of the cell pyroptosis rate. However, KAT2A overexpression abolished HOXA5 overexpression‐mediated suppression of pyroptosis in LPS‐induced ATII cells (Figure 5G). Taken together, KAT2A overexpression reversed HOXA5 overexpression‐mediated suppression of pyroptosis in LPS‐induced ATII cells.

### 4.6 HOXA5 Upregulation Inhibited the KAT2A/NEK7 Axis to Improve ALI in Mice

**FIGURE 6:**
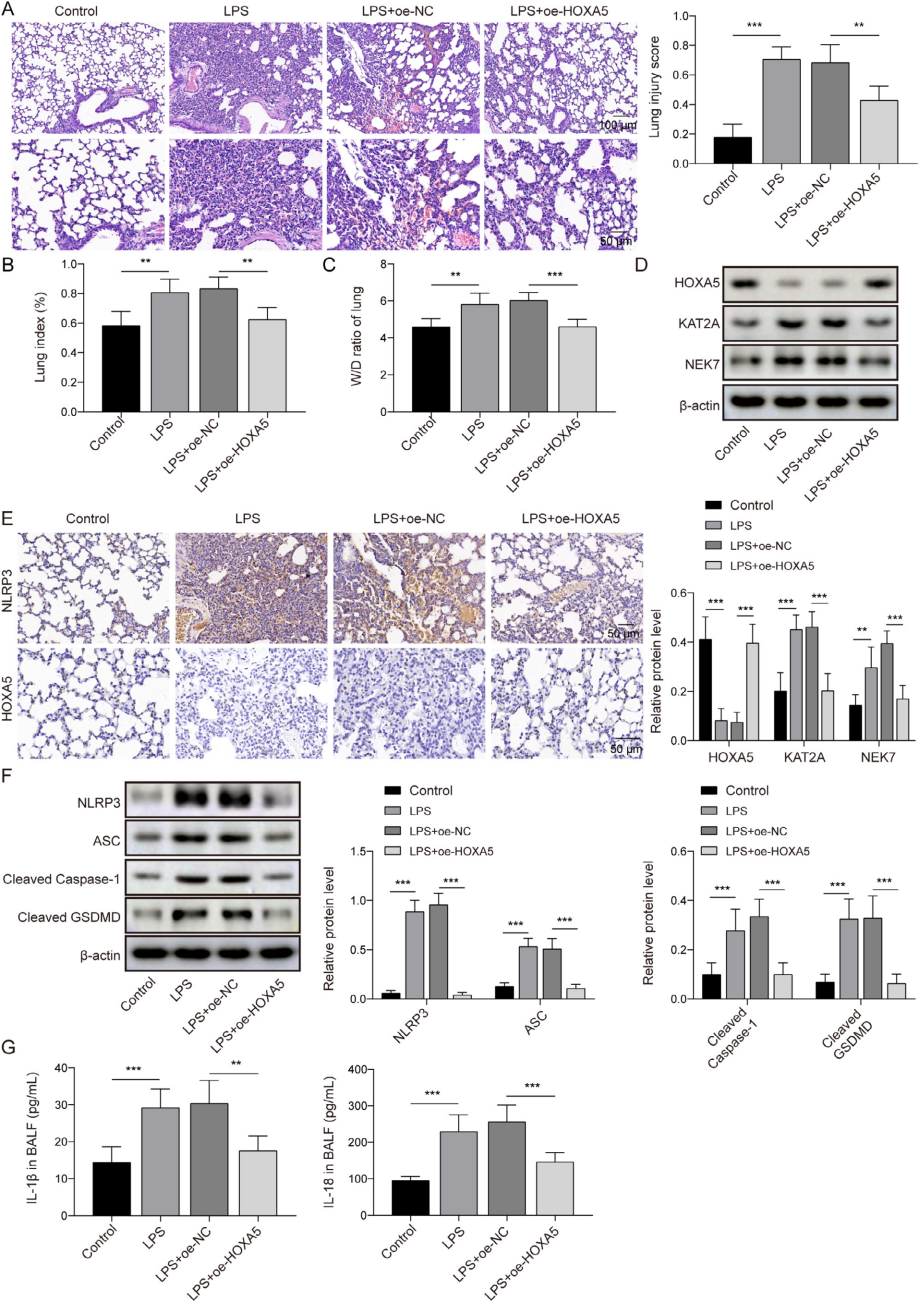
Homeobox A5 (HOXA5) upregulation inhibited the lysine acetyltransferase 2A KAT2A/NEK7 axis to improve ALI in mice. The lipopolysaccharide (LPS)‐induced ALI mouse model was established, and mice were treated with a lentiviral vector carrying oe‐HOXA5 to overexpress HOXA5 5 days before LPS induction. (A) Pulmonary tissue pathology was evaluated using HE staining and lung injury scores. (B) Lung index and (C) lung W/D ratios were measured. (D) HOXA5, KAT2A, and NEK7 expression levels were examined using Western blot. (E) HOXA5 and NLRP3 expression levels were detected using IHC assay. (F) NLRP3, ASC, Cleaved Caspase‐1, and Cleaved GSDMD levels were examined using Western blot. (G) IL‐1β and IL‐18 levels in bronchoalveolar lavage fluid were detected using enzyme‐linked immunosorbent assay (ELISA). *p < 0.05, **p < 0.01, ***p < 0.001.

To investigate the role of HOXA5 in LPS‐induced ALI in mice, animals were treated with lentiviral vectors carrying oe‐HOXA5 prior to LPS induction. As depicted in Figure 6A, HE staining showed that LPS treatment caused extensive alveolar structural damage and a significant infiltration of inflammatory cells. In contrast, the degree of lung tissue damage was significantly reduced in mice overexpressing HOXA5. Additionally, LPS exposure led to a significant increase in lung index and lung W/D ratio. However, HOXA5 overexpression effectively decreased the levels of both indicators (Figure 6B,C). LPS treatment resulted in the downregulation of HOXA5 expression and upregulation of KAT2A and NEK7 expression, which were compromised by HOXA5 upregulation (Figure 6D). Meanwhile, IHC revealed that LPS downregulated HOXA5 and upregulated NLRP3 expression. These patterns were reversed by HOXA5 overexpression (Figure 6E). Further detection of pyroptosis‐related proteins by Western blot revealed that LPS increased NLRP3, ASC, Cleaved Caspase‐1, and Cleaved GSDMD levels. HOXA5 overexpression effectively reversed these changes (Figure 6F). Additionally, ELISA detection of inflammatory factors in bronchoalveolar lavage fluid showed that LPS promoted the secretion of IL‐1β and IL‐18, which was suppressed by HOXA5 overexpression (Figure 6G). Taken together, these results suggest that HOXA5 upregulation suppresses pyroptosis and inflammation in the lungs, thereby ameliorating LPS‐induced ALI in mice, likely through inhibition of the KAT2A/NEK7 axis.

## 5 Discussion

Although there have been rapid advancements in treatment strategies and methods for ALI in recent years, the mortality rate among patients remains high. As previously documented, the in‐hospital mortality rate for patients with mild ALI is 34.9%, whereas that for those with moderate‐to‐severe ALI approaches 50% [22]. Therefore, understanding the pathological mechanisms underlying ALI, along with crucial molecular regulations implicated in disease progression, is of profound significance. Pyroptosis in ATII cells plays a critical role in ALI [23, 24]. For example, LL‐37 suppressed pyroptosis in ATII cells to ameliorate sepsis‐induced ALI [24]. Here, we investigated the molecular mechanisms underlying pyroptosis in LPS‐induced ATII cells. Our findings revealed that the upregulation of HOXA5 inhibited KAT2A, preventing the recruitment of H3K9ac/H3K27ac to the NEK7 promoter region. This, in turn, decreased NEK7 expression and suppressed pyroptosis in ALI models. Thus, targeting HOXA5 may offer a promising therapeutic strategy for treating ALI.

HOXA5 plays important roles in multiple diseases [18, 25]. Weina Cao et al. demonstrated that HOXA5 mitigates adipocyte inflammation and promotes adipose tissue browning by inhibiting the TNC/TLR4/NF‐κB inflammatory axis and activating the BMP4/Smad1 pathway, suggesting HOXA5 could be a target for inflammatory diseases [26]. Additionally, SIRT5‐mediated desuccinylation of HOXA5 enhances its expression, which in turn suppresses ferroptosis and ameliorates sepsis‐induced lung injury [8]. In this study, we constructed both in vitro and in vivo models and found a reduction in HOXA5 expression in ATII cells and in mice. Moreover, dexmedetomidine pretreatment affected HOXA5 expression, effectively inhibiting propofol‐induced pyroptosis in primary hippocampal neurons [27]. METTL14 promotes WNK1 expression by mediating N6‐methyladenosine (m6A) modification of HOXA5, thereby enhancing HOXA5 expression and inhibiting NLRP3‐dependent pyroptosis to alleviate osteoporosis [9]. Notably, whether HOXA5 regulates pyroptosis in ATII cells induced by ALI has not been previously determined. Our findings in this study reveal that HOXA5 overexpression significantly attenuated LPS‐induced pyroptosis in both ATII cells and mice.

NEK7 is a highly conserved serine/threonine kinase involved not only in the mammalian cell cycle and mitosis, but also in a plethora of other biological processes [10]. Increasing evidence suggests that NEK7 facilitates the activation of the NLRP3 inflammasome, thereby mediating pyroptosis in various diseases, including ALI [28]. As previously reported, the NLRP3 inflammasome contributes to the production of inflammatory mediators and the infiltration of immune cells, leading to increased permeability of the pulmonary capillary membrane. This process ultimately results in pulmonary edema and lung tissue damage, exacerbating conditions like ventilator‐induced lung injury [29]. Moreover, in cyclic stretch (CS)‐stimulated mouse lung epithelial cells and in rats with ventilator‐induced lung injury, the interaction between NEK7 and NLRP3 was enhanced, while NEK7 knockdown reduced this interaction and inactivated the NLRP3 inflammasome [29]. Similarly, in sepsis‐induced ALI, NEK7 expression was elevated in alveolar macrophages, and WHSC1 knockdown inhibited NEK7 expression, thereby weakening NEK7‐mediated activation of the NLRP3 inflammasome and inhibiting pyroptosis in alveolar macrophages [15]. In our study, we confirmed the direct interactions between NEK7 and NLRP3. Furthermore, NEK7 overexpression activated the NLRP3 inflammasome, as indicated by elevated expression of pyroptosis‐related proteins, including NLRP3, ASC, Cleaved Caspase‐1, and Cleaved GSDMD. Interestingly, HOXA5 overexpression decreased NEK7 expression in LPS‐induced ATII cells and mice, and NEK7 overexpression reversed the inhibitory effects of HOXA5 on pyroptosis in these models.

In the present study, we demonstrated that HOXA5 negatively regulates NEK7 expression in LPS‐induced ATII cells. Although HOXA5, as a transcription factor, is expected to bind directly to the NEK7 promoter and regulate NEK7 expression, our ChIP results suggested no direct interaction between HOXA5 and the NEK7 promoter. Using the Cistrome database, we predicted that the NEK7 promoter might be subject to histone modifications, potentially explaining NEK7 elevated expression in LPS‐induced ATII cells. Based on this, we hypothesized that HOXA5 indirectly regulates NEK7 expression by regulating specific target genes, which then affect the epigenetic landscape of NEK7. KAT2A, an acetyltransferase in the GNAT family [30], is a key enzyme in the regulation of gene transcription. KAT2A catalyzes the acetylation of lysine residues on histones and free proteins [31]. Moreover, KAT2A facilitates the transcription of target genes by mediating H3K9ac and the acetylation of lysine at position 36 of histone H3 (H3K36ac) and H3K27ac [32, 33, 34]. In our study, KAT2A was elevated in LPS‐induced ATII cells, and KAT2A elevated NEK7 expression by enriching H3K9ac and H3K27ac in the NEK7 promoter region. These findings indicate that the elevated expression of NEK7 in LPS‐induced ATII cells is regulated by KAT2A. Furthermore, we predicted that HOXA5 could bind to the KAT2A promoter and experimentally confirmed this interaction. Previous studies have shown that HOXA5 binds to the promoter region of target genes, inhibiting their transcriptional activity and reducing their expression [18, 35]. For example, HOXA5 suppresses the transcription of Jag1 and mitigates the progression of renal fibrosis [18], and similarly, it downregulates SLC7A11 to enhance ferroptosis in cervical squamous cell carcinoma cells [35]. Herein, the transcriptional activity and expression of KAT2A in LPS‐induced ATII cells were also inhibited by HOXA5 overexpression. This inhibition led to decreased enrichment of H3K9ac and H3K27ac in the NEK7 promoter region, thereby reducing NEK7 expression.

In conclusion, our study reveals that low expression of HOXA5 promoted KAT2A transcription, then promoted NEK7 expression by recruiting H3K9ac/H3K27ac to the NEK7 promoter region, ultimately driving NLRP3‐mediated pyroptosis in ALI models. Thus, HOXA5 plays a critical role in the pathogenesis of ALI by inhibiting NEK7‐mediated pyroptosis in alveolar epithelial cells. This molecular mechanism not only enhances our understanding of the pathological mechanisms underlying ALI but also provides new avenues for therapeutic interventions. Based on these findings, small‐molecule inhibitors targeting HOXA5 are expected to become a promising strategy for the treatment of ALI.

## Conflicts of Interest

The authors declare no conflicts of interest.

## Supporting information


**Figure S1:** Identification of ATII cells. ATII cells were identified through IF staining of SP‐A.

## Data Availability

Data sharing is not applicable to this article as no datasets were generated or analyzed during the current study.
