# Supplementary figures and images for: HOXA5 Suppresses NEK7‐Mediated Alveolar Epithelial Pyroptosis in Acute Lung Injury by Transcriptionally Inhibiting KAT2A
Source: Kaohsiung J Med Sci. 2025 Sep 25;42(3):e70109. doi: 10.1002/kjm2.70109 (PMC12955922; doi:10.1002/kjm2.70109)

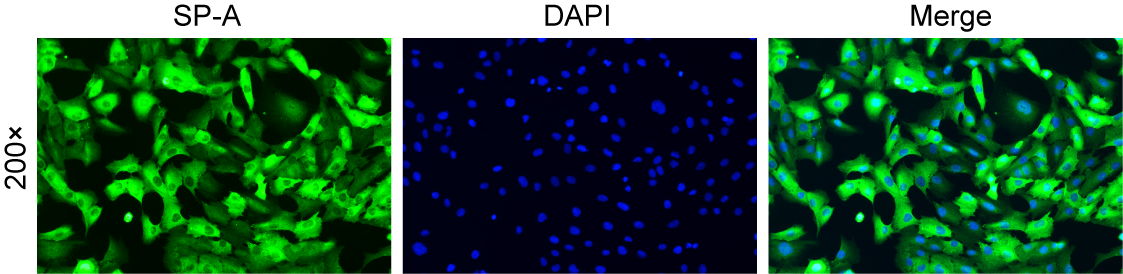

Supplement: Supplementary file 1 — Figure S1: Identification of ATII cells. ATII cells were identified through IF staining of SP‐A. [file KJM2-42-e70109-s001.tif]
